# Assessment of the Accuracy of Firearm Injury Intent Coding at 3 US Hospitals

**DOI:** 10.1001/jamanetworkopen.2022.46429

**Published:** 2022-12-13

**Authors:** Matthew Miller, Deborah Azrael, Ravali Yenduri, Catherine Barber, Andrew Bowen, Erin MacPhaul, Stephen J. Mooney, Li Zhou, Eric Goralnick, Ali Rowhani-Rahbar

**Affiliations:** 1 Department of Health Sciences, Bouvé College of Health Sciences, Northeastern University, Boston, Massachusetts; 2 Harvard Injury Control Research Center, Harvard T. H. Chan School of Public Health, Boston, Massachusetts; 3 Department of Emergency Medicine, Brigham and Women’s Hospital, Harvard Medical School, Boston, Massachusetts; 4 Center for Surgery and Public Health, Brigham and Women’s Hospital, Boston, Massachusetts; 5 Firearm Injury and Policy Research Program, University of Washington, Seattle; 6 Department of Epidemiology, School of Public Health, University of Washington, Seattle

## Abstract

**Importance:**

The absence of reliable hospital discharge data regarding the intent of firearm injuries (ie, whether caused by assault, accident, self-harm, legal intervention, or an act of unknown intent) has been characterized as a glaring gap in the US firearms data infrastructure.

**Objective:**

To use incident-level information to assess the accuracy of intent coding in hospital data used for firearm injury surveillance.

**Design, Setting, and Participants:**

This cross-sectional retrospective medical review study was conducted using case-level data from 3 level I US trauma centers (for 2008-2019) for patients presenting to the emergency department with an incident firearm injury of any severity.

**Exposures:**

Classification of firearm injury intent.

**Main Outcomes and Measures:**

Researchers reviewed electronic health records for all firearm injuries and compared intent adjudicated by team members (the gold standard) with *International Classification of Diseases*, *Ninth* and *Tenth Revision, Clinical Modification* (*ICD-9-CM* and *ICD-10-CM*) codes for firearm injury intent assigned by medical records coders (in discharge data) and by trauma registrars. Accuracy was assessed using intent-specific sensitivity and positive predictive value (PPV).

**Results:**

Of the 1227 cases of firearm injury incidents seen during the *ICD-10-CM* study period (October 1, 2015, to December 31, 2019), the majority of patients (1090 [88.8%]) were male and 547 (44.6%) were White. The research team adjudicated 837 (68.2%) to be assaults. Of these assault incidents, 234 (28.0%) were *ICD* coded as unintentional injuries in hospital discharge data. These miscoded patient cases largely accounted for why discharge data had low sensitivity for assaults (66.3%) and low PPV for unintentional injuries (34.3%). Misclassification was substantial even for patient cases described explicitly as assaults in clinical notes (sensitivity of 74.3%), as well as in the *ICD-9-CM* study period (sensitivity of 77.0% for assaults and PPV of 38.0% for unintentional firearm injuries). By contrast, intent coded by trauma registrars differed minimally from researcher-adjudicated intent (eg, sensitivity for assault of 96.0% and PPV for unintentional firearm injury of 93.0%).

**Conclusions and Relevance:**

The findings of this cross-sectional study underscore questions raised by prior work using aggregate count data regarding the accuracy of *ICD*-coded discharge data as a source of firearm injury intent. Based on our observations, researchers and policy makers should be aware that databases drawn from hospital discharge data (most notably, the Nationwide Emergency Department Sample) cannot be used to reliably count or characterize intent-specific firearm injuries.

## Key Points

**Question:**

Do routinely collected hospital data accurately identify whether individuals presenting to emergency departments with gunshot wounds were shot as a result of an assault, an accident, an act of deliberate self-harm, or during legal intervention?

**Findings:**

In this cross-sectional study of 1227 patients, coding accuracy of firearm injury incidents at 3 level I US trauma centers was assessed using case-level data for 2008 to 2019. The results showed that 28% of all emergency department visits for assault-related firearm injuries were miscoded as firearm accidents in hospital discharge data.

**Meaning:**

These findings suggest that the predominant misclassification problem with firearm injury intent in hospital discharge data is assaults miscoded as accidents, raising questions about studies that have used discharge data to study firearm injury by intent.

## Teaser

This cross-sectional study uses case-level electronic health records for 2008 to 2019 from 3 level I US trauma centers to assess intent coding accuracy in hospital data used for firearm injury surveillance.

## Introduction

In 2020, 45 222 individuals were killed by firearms in the US—5515 more than in 2019.^1^ This 1-year increase was the largest in US history and was primarily attributable to increases in homicides and suicides (4970 and 351 more in 2020, respectively), with smaller increases in deaths from accidents or legal intervention and in firearm deaths for which intent could not be determined.^1^ Reliable data on the corresponding number of intent-specific nonfatal firearm injuries (ie, those caused by assault, self-harm, accident, legal intervention, or of undetermined intent), however, are lacking because hospital discharge data, despite being the most comprehensive source of information about individuals with gunshot injuries presenting to US emergency departments (EDs), frequently misclassify firearm injury intent.^2,3,4,5^

An expert panel recently characterized this coding problem as a glaring gap in the US firearms data infrastructure.^5^ To better understand the nature of this problem, we reviewed electronic health records (EHRs) for firearm injury encounters at 3 level I trauma centers from 2008 to 2019 and compared researcher-adjudicated intent for each incident with the intent indicated by the *International Classification of Diseases* (*ICD*) code assigned by the hospital medical records coder (ie, in discharge data).^4^ Because prior work with aggregate count data indicates that discharge data underestimate the number of firearm assaults and overestimate the number of unintentional firearm injuries,^2,4,6^ we examined medical record narratives to identify whether patterns of misclassification reflected (1) well-defined and potentially remediable definitional misconceptions (eg, mistakenly thinking that a gunshot wound sustained by a bystander during an assault should be coded as an unintentional injury), (2) a tendency to default to unintentional intent when narrative information was suggestive of but did not explicitly identify an intent (eg, a patient with multiple gunshot injuries reports walking down the street when he heard shots and realized he was wounded), or (3) a broader tendency to code firearm injuries as unintentional even when another intent, such as assault, was explicitly asserted or unambiguously suggested in the narrative (eg, the shooting was described as resulting from a robbery).

We also assessed how trauma registrars coded firearm injury intent. Trauma registrars are allied health professionals who focus exclusively on traumatic injuries and are explicitly charged with recording detailed information about the circumstances that led to the injury, including intent. By contrast, medical records coders are hired for billing purposes to review charts and code diagnoses, procedures, and a nonbillable “external cause of injury” *ICD* code (for patients with injury) specifying the mechanism (eg, firearm, knife) and the intent that caused the injury. Trauma centers are required to maintain a registry of data collected by trauma registrars that, although not a population-based sample of ED visits, had been used to study intent-specific firearm injuries.^6,7,8,9,10,11,12^

## Methods

This retrospective cross-sectional study was approved by the Massachusetts General Brigham (MGB) Research Ethics Board and the University of Washington Human Subjects Division, with waiver of informed consent from study participants owing to secondary use of EHR data. The study followed the Strengthening the Reporting of Observational Studies in Epidemiology (STROBE) reporting guideline.

### Patient Population and Data Sources

We used data from 3 hospitals: Brigham and Women’s Hospital (BWH) and Massachusetts General Hospital, which belong to a single hospital system (MGB), for 2008 to 2019, and the University of Washington–managed Harborview Medical Center (HMC) for March 20, 2016, to January 31, 2018. Eligible incidents were those in which the medical records coder included a firearm-related e-code in any e-code or diagnosis field. For all records before September 30, 2015, *International Classification of Diseases, Ninth Revision, Clinical Modification* (*ICD-9-CM*) codes were used (E965 [0.0-0.4, 0.9], E979.4, E955 [0.0-0.9], E922 [0.0-0.3, 0.8, 0.9], E985 [0.0-0.4], and E970). For October 1, 2015, and all time points afterward, *International Classification of Diseases, Tenth Revision, Clinical Modification* (*ICD-10-CM*) codes were used (W320XX-WW321XX, W330XX-W3309X, W3309X-W3313X, W3319X, W3400X, W3409X-W3410X, W3419X, X72XXX, X730XX-X732XX, X738XX-X739XX, X748XX-X749XX, Y384X1-Y384X3, X93XXX, X940XX-X942XX, X948XX-X949XX, X958XX-X959XX, Y22XXX, Y230XX-Y233XX, Y238XX-Y239XX, Y248XX-Y249XX, Y35001-Y35003, Y35009, Y35011-Y35013, Y35019, Y35021-Y35023, Y35029, Y35031-Y35033, Y35039, Y35091-Y35093, and Y35099).

### Researcher-Adjudicated Intent

Members of the research team (R.Y. and E.M. for MGB and A.B. for HMC) reviewed the medical record for each eligible patient case. For all incidents that, on review, met our case definition, reviewers assigned an injury intent using a detailed coding manual developed for the project (eAppendix in Supplement 1). Firearm injury incidents met our case definition if the injured person made an initial presentation to 1 of our EDs for an injury resulting from a traditional projectile fired from a firearm (eg, not a BB gun injury, a blunt force injury from being struck by a firearm, or an injury from a rubber bullet or beanbag shot). If a person suffered a new gunshot injury over the project period, this new injury was considered a new incident firearm injury. Visits for follow-up care or late effects were not, by definition, treated as new injuries.

The intent categories assigned by reviewers were assault, accidental, intentional self-injury, legal intervention, and undetermined. We differentiated cases of undetermined intent owing to a lack of information from those for which there was credible conflicting information about intent. In addition to assigning an intent to each case, researchers coded information as follows: the circumstances of the injury, the nature of the injury (including the number of gunshot wounds [single or multiple]), the wound location, and the activity of the person wounded at the time of the incident. Our coding manual drew on definitions and rules for coding intent from codebooks for the National Violent Death Reporting System,^13^ which were then refined iteratively by team members as we reviewed cases. To facilitate review and allow for subsequent analyses, researchers also extracted all intent-related phrases from each case as free text.

For 111 cases (9.0%), at least 2 study team members coded the same case via review of the medical record. Investigators chose differing intents in 6 cases (4.5%). Disagreements about the intent of an injury were resolved by the project team. For any cases in which the discrepancy was the result of ambiguity in guidance, the coding manual was changed to resolve the ambiguity, and all already-coded cases that might have been affected by the clarification were reviewed and updated. Researcher-adjudicated coding in our final data set reflected the rules specified in the final version of the codebook.

### Data Linkage

Data extracted from the EHR (eg, our coded data, including verbatim extracts) were linked by medical record number and admission date to trauma registry intent data (ie, the e-code assigned by trauma registrars). At BWH and HMC, the trauma registry included all ED-treated firearm injuries, regardless of severity or admission status. At MGB, cases coded by trauma registrars excluded firearm injury incidents in which the patient was treated in the ED and “released” (ie, sent home without being admitted or observed for >24 hours in the ED). The EHR data were also linked to administrative data to retrieve patients’ self-reported (or informant-reported) demographics. Administrative data on self-reported race and ethnicity (Black, Hispanic, White, other racial or ethnic minority group [including American Indian or Alaska Native, Asian, Middle Eastern or North African, Native Hawaiian or other Pacific Islander, and multiple races or ethnicities], or unknown or missing) were used to illustrate how the burden of intent-specific firearm injury differed depending on who assigned the intent (ie, medical records coders, trauma registrars, or researchers).

### Statistical Analysis

We calculated intent-specific sensitivity and positive predictive value (PPV) in discharge data and trauma registry data using researcher-adjudicated intent as the gold-standard intent for each patient case. We also assessed the free-text phrases extracted for each case to distinguish incidents in which the medical record contained explicit language indicating intent (eg, an assault in which the shooter was explicitly called an assailant, or where an altercation clearly led to the injury) from incidents where researcher-adjudicated intent was based only on circumstantial default criteria laid out in the coding manual (eg, an assault based on the incident involving multiple people wounded, or text indicating the patient reported only that he had been shot while walking down the street).

Our primary analyses focused on *ICD-10-CM* coded data (and comprised cases for October 1, 2015, to December 31, 2019). We also examined *ICD-9-CM* coded cases (January 1, 2008, to September 30, 2015, available only at MGB) to assess the extent to which misclassification patterns identified in *ICD-10-CM* data were present before the explicit change in official intent-related default guidance that accompanied the switch from *ICD-9-CM* (ie, default to undetermined intent if injury intent is not described in the chart) to *ICD-10-CM* (ie, default to accident if intent of the injury is not described in the chart).^2^

## Results

In this cross-sectional study, 1227 patient cases with firearm injury presented at 1 of 3 study sites during the *ICD-10-CM* period. Of these individuals, 1090 were male (88.8%) and 136 were female (11.1%; sex was not reported for 1 patient); 854 (69.6%) were aged 18-39 years and 303 (24.7%) were aged 40 years or older (Table 1). With regard to race, 432 patients (35.2%) identified as Black, 547 (44.6%) as White, and 203 (16.5%) as another racial or ethnic minority group; data on race were unknown or missing for 66 (5.4%). Features of intent-specific firearm injuries differed when patient cases were sorted by researcher-adjudicated intent vs medical records coder *ICD*-coded intent. For example, patient cases identified as unintentional by the research team were older, less likely to have died, and less likely to have been injured in the head, face, or neck than those *ICD* coded as unintentional. For patient cases in which information was too sparse for researchers to assign an intent other than undetermined, a large proportion died (12 of 55 [21.8%]) and the distribution of characteristics closely resembled that of the researcher-adjudicated assault cases.

**Table 1.** Patient Demographic and Firearm Injury Characteristics by Intent Assigned by Medical Records Coders (Using *ICD-10-CM* Codes) vs Researcher-Adjudicated Intent

Characteristic	No. of patients (%) (N = 1227)	Intent, No. of cases (%)									
		Unintentional		Assault^a^		Legal intervention		Self-harm		Undetermined^b^	
		*ICD-10-CM* (n = 432)	Researcher (n = 168)	*ICD-10-CM* (n = 581)	Researcher (n = 837)	*ICD-10-CM* (n = 32)	Researcher (n = 43)	*ICD-10-CM* (n = 119)	Researcher (n = 124)	*ICD-10-CM* (n = 63)	Researcher (n = 55)
Sex^c^											
Female	136 (11.1)	39 (9.0)	18 (10.7)	68 (11.7)	91 (10.9)	3 (9.4)	3 (7.0)	18 (15.1)	16 (12.9)	8 (12.7)	8 (14.5)
Male	1090 (88.8)	393 (91.0)	150 (89.3)	512 (88.1)	745 (89.0)	29 (90.6)	40 (93.0)	101 (84.9)	108 (87.1)	55 (87.3)	47 (85.5)
Age, y											
0-17	70 (0.1)	21 (4.9)	8 (4.8)	34 (5.9)	51 (6.1)	2 (6.3)	2 (4.7)	7 (5.9)	9 (7.3)	6 (9.5)	0 (0.0)
18-39	854 (69.6)	305 (70.6)	100 (59.5)	431 (74.2)	627 (74.9)	20 (62.5)	28 (65.1)	57 (47.9)	60 (48.4)	41 (65.1)	39 (70.9)
>40	303 (24.7)	106 (24.5)	60 (35.7)	116 (20.0)	159 (19.0)	10 (31.3)	13 (30.2)	55 (46.2)	55 (44.4)	16 (25.4)	16 (29.1)
Race											
Black	432 (35.2)	137 (31.7)	23 (13.7)	252 (43.4)	372 (44.4)	7 (21.9)	6 (14.0)	10 (8.4)	11 (8.9)	26 (41.3)	20 (36.4)
White	547 (44.6)	213 (49.3)	130 (77.4)	193 (33.2)	267 (31.9)	21 (65.6)	29 (67.4)	95 (79.8)	99 (79.8)	25 (39.7)	22 (40.0)
Other^d^	203 (16.5)	67 (15.5)	11 (6.5)	116 (20.0)	169 (20.2)	3 (9.4)	5 (11.6)	8 (6.7)	7 (5.6)	9 (14.3)	11 (20.0)
Unknown or missing	66 (5.4)	22 (5.1)	5 (3.0)	29 (5.0)	43 (5.1)	2 (6.3)	4 (9.3)	10 (8.4)	11 (8.9)	3 (4.8)	3 (5.5)
Ethnicity											
Hispanic	247 (20.1)	84 (19.4)	21 (12.5)	141 (24.3)	202 (24.1)	4 (12.5)	8 (18.6)	7 (5.9)	6 (4.8)	11 (17.5)	10 (18.2)
Other^e^	922 (75.1)	326 (75.5)	145 (86.3)	416 (71.6)	597 (71.3)	27 (84.4)	33 (76.7)	103 (86.6)	107 (86.3)	50 (79.4)	40 (72.7)
Unknown or missing	58 (4.7)	22 (5.1)	2 (1.2)	24 (4.1)	38 (4.5)	1 (3.1)	2 (4.7)	9 (7.6)	11 (8.9)	2 (3.2)	5 (9.1)
Patient disposition											
Admitted to ED	18 (1.5)	4 (0.9)	0 (0.0)	13 (2.2)	16 (1.9)	1 (3.1)	1 (2.3)	0 (0.0)	0 (0.0)	0 (0.0)	1 (1.8)
Died	155 (12.6)	36 (8.3)	7 (4.2)	44 (7.6)	66 (7.9)	5 (15.6)	8 (18.6)	54 (45.4)	62 (50.0)	16 (25.4)	12 (21.8)
Treated and released	307 (25.0)	113 (26.2)	45 (26.8)	176 (30.3)	243 (29.0)	2 (6.3)	4 (9.3)	0 (0.0)	0 (0.0)	16 (25.4)	15 (27.3)
Transferred	7 (0.01)	1 (0.2)	1 (0.6)	3 (0.5)	3 (0.4)	0 (0.0)	0 (0.0)	3 (2.5)	3 (2.4)	0 (0.0)	0 (0.0)
Admitted to the hospital	740 (60.3)	278 (64.4)	115 (68.5)	345 (59.4)	509 (60.8)	24 (75.0)	30 (69.8)	62 (52.1)	59 (47.6)	31 (49.2)	27 (49.1)
Mean (SD) length of stay, d											
Treated and released	NA	0.3 (0.7)	0.3 (0.4)	0.3 (0.7)	0.3 (0.7)	0.0 (0.0)	0.0 (0.0)	0.0 (0.0)	0.0 (0.0)	0.2 (0.5)	0.5 (1.6)
Admitted to the hospital	NA	7.2 (10.4)	5.3 (8.5)	11.3 (16.7)	10.2 (15.2)	21.9 (25.5)	20.5 (22.9)	15.7 (10.9)	15.7 (10.9)	5.9 (7.1)	8.8 (15.1)
Multiple people wounded											
No	1070 (87.2)	387 (89.6)	162 (96.4)	482 (83.0)	698 (83.4)	29 (90.6)	38 (88.4)	115 (96.6)	118 (95.2)	57 (90.5)	54 (98.2)
Yes	157 (12.8)	45 (10.4)	6 (3.6)	99 (17.0)	139 (16.6)	3 (9.4)	5 (11.6)	4 (3.4)	6 (4.8)	6 (9.5)	1 (1.8)
Body location injured											
Head	159 (13.0)	39 (9.0)	5 (3.0)	47 (8.1)	73 (8.7)	13 (40.6)	4 (9.3)	55 (46.2)	68 (54.8)	14 (22.2)	9 (16.4)
Face	121 (9.9)	36 (8.3)	9 (5.4)	45 (7.7)	68 (8.1)	0 (0.0)	2 (4.7)	37 (31.1)	37 (29.8)	3 (4.8)	5 (9.1)
Neck	44 (3.6)	13 (3.0)	1 (0.6)	25 (4.3)	36 (4.3)	1 (3.1)	2 (4.7)	3 (2.5)	3 (2.4)	2 (3.2)	2 (3.6)
Upper extremity	305 (24.9)	139 (32.2)	63 (37.5)	137 (23.6)	217 (25.9)	14 (43.8)	18 (41.9)	2 (1.7)	1 (0.8)	13 (20.6)	7 (12.7)
Back	29 (2.4)	8 (1.9)	0 (0.0)	21 (3.6)	25 (3.0)	0 (0.0)	2 (4.7)	0 (0.0)	0 (0.0)	0 (0.0)	2 (3.6)
Chest	230 (17.7)	66 (15.3)	12 (7.1)	122 (21.0)	176 (21.0)	15 (46.9)	21 (48.8)	15 (12.6)	13 (10.5)	12 (19.1)	8 (14.5)
Abdomen	213 (17.4)	70 (16.2)	16 (9.5)	113 (19.4)	167 (20.0)	10 (31.3)	15 (34.9)	9 (7.6)	6 (4.8)	11 (17.5)	9 (16.4)
Lower extremity	476 (38.8)	164 (38.0)	78 (46.4)	265 (45.6)	366 (43.7)	11 (34.4)	14 (32.6)	6 (5.0)	1 (0.8)	26 (41.3)	17 (30.9)

Abbreviations: ED, emergency department; *ICD-10-CM*, *International Classification of Diseases, Tenth Revision, Clinical Modification*; NA, not applicable.

^a^ Of 157 incidents, 5 were assigned to assaults on the basis of multiple individuals having been wounded.

^b^ Eight patient cases with multiple *ICD-10-CM* external cause codes are included.

^c^ Data on sex were classified as “other” for 1 individual.

^d^ Includes American Indian or Alaska Native, Asian, Middle Eastern or North African, Native Hawaiian or Other Pacific Islander, or mulitple races or ethnicities.

^e^ Includes patients who did not identify as Hispanic or Latino.

The research team observed that of the 1227 firearm injury incidents, 837 (68.2%) resulted from assaults, 168 (13.5%) were unintentionally inflicted injuries, 124 (9.9%) were acts of deliberate self-harm, and 43 (3.4%) were legal intervention incidents. For 55 patient cases, it was not possible to identify an intent (4.4%); record review identified credible conflicting information about intent for 19 (34.5%) of these cases (Table 2). According to *ICD*-coded discharge data, 581 (47.4%) of the 1227 cases were assaults and 432 (35.2%) were unintentional injuries, with less dramatic discrepancies between discharge data and researcher-adjudicated intent for the remaining intent-specific injury types.

**Table 2.** Firearm Injury Intent in *ICD-10-CM* Hospital Discharge Data Compared With Researcher-Adjudicated Intent, From October 1, 2015, to December 31, 2019

Researcher-adjudicated intent	No. of patient cases with *ICD*-coded intent in hospital discharge data							*ICD* intent-specific sensitivity, %^c^
	Unintentional	Assault	Legal intervention	Suicide/self-harm	Undetermined^a^	Multiple intents^b^	Total	
Unintentional	148	8	0	4	8	0	168	88.1
Assault	234	555	2	0	40	6	837	66.3
Legal intervention	9	2	30	1	0	1	43	69.8
Suicide/self-harm	9	0	0	111	4	0	124	89.5
Undetermined	32	16	0	3	3	1	55	5.0
Total	432	581	32	119	55	8	1227	NA
Intent-specific PPV of *ICD*-coded data, %^d^	34.3	95.5	93.8	93.3	5.5	NA	NA	NA

Abbreviations: *ICD-10-CM*, *International Classification of Diseases*, *Tenth Revision, Clinical Modification*; NA, not applicable; PPV, positive predictive value.

^a^ Of these patient cases, 36 were undetermined due to a lack of information and 19 were undetermined because of credible conflicting information.

^b^ Eight cases were coded with 2 or more *ICD* codes with conflicting intents (eg, assault and undetermined).

^c^ Among cases in each intent category (according to the researcher-adjudicated standard), we report the percentage of cases that were coded to that intent by medical records coders. Sensitivity is based only on the universe of firearm injuries that we identified via firearm e-codes (ie, we did not attempt to identify firearm injury cases that were outside the range of all e-coded firearm injuries).

^d^ Among cases in each intent category (according to the medical records coders), we report the percentage that were true positives for that intent category according to researcher-adjudicated attribution of intent. No cases fell into the *ICD* “terrorism” or “operations of war” intent categories.

Table 2 shows that the predominant intent-related misclassification problem with discharge data was miscoding intentional firearm injuries as accidents. For example, 234 of 837 assaults (28.0%) were misclassified in discharge data as unintentional injuries, as were 9 of 43 (20.9%) legal intervention injuries. Accordingly, the PPV for unintentional firearm injuries in discharge data was low (34.3%), as was the sensitivity for assaults (66.3%). This misclassification pattern was also apparent, although less pronounced, in the *ICD-9-CM* data (eg, PPV for unintentional firearm injuries was 38.0%, and the sensitivity for assaults was 77.0%; eTable 1 in Supplement 1). The miscoding patterns we observed in primary analyses were apparent in stratified analyses (ie, similar patterns were evident at each study site, for cases that proved fatal, for nonfatal cases, for those treated and released from the ED, and for those admitted as inpatients; eTable 1 in Supplement 1).

Even among the 52.4% of researcher-adjudicated assaults in which the medical narrative explicitly indicated that the shooting was an act of interpersonal violence (eg, a drive-by shooting, an act of domestic violence, or a gang-related attack), misclassification of intent by medical coders was substantial (Table 3). Overall, discharge data identified 22.0% of such incidents as unintentional injuries. eTable 2 in Supplement 1 illustrates how subtypes of unambiguous assaults were coded in discharge data: 21.1% of assaults clearly described in the medical record as drive-by shootings were *ICD* coded as unintentional firearm injuries in discharge data, as were 17.9% of those in which the word “assailant” was used to describe the shooter and 18.4% of those in which the shooting occurred during an altercation.

**Table 3.** Researcher-Adjudicated Assaults Sorted by Type of Evidence of Intent and by *ICD*-Coded Intent^a^

Researcher-adjudicated assaults	*ICD*-coded intent in discharge data			
	Total	Assault	Accident	All other
Type of evidence of assault intent				
Explicit language indicating an interpersonal circumstance explicitly noted as violence related (robbery, altercation, attack, drive-by, gang related, domestic violence, community violence, revenge, etc)	255 (30.5)	183 (71.7)	63 (24.8)	9 (3.5)
Shooter referred to as an assailant, attacker, or similar	97 (11.6)	74 (75.8)	17 (17.9)	6 (6.3)
Both (circumstance and shooter information indicate violence related)	87 (10.4)	70 (80.5)	16 (18.4)	1 (1.1)
Subtotal	439 (52.4)	326 (74.3)	97 (22.0)	16 (3.7)
Assault inferred from circumstantial evidence only (multiple people wounded, multiple gunshots, activity or location known at time of shooting [eg, walking down the street when shot, standing on corner when shot])	398 (47.6)	233 (58.5)	139 (34.9)	26 (6.6)
Total	837 (100)	559 (66.8)	236 (28.2)	42 (5.0)

Abbreviation: *ICD*, *International Classification of Diseases*.

^a^ Data are presented as No. (%) of cases.

Intent identified by trauma registrars, in contrast, seldom differed from intent assigned by the research team (Table 4); for example, sensitivity for assault was 96.0% and the PPV for unintentional firearm injury was 93.0%. Close agreement between researcher and trauma registrar–coded cases was reflected not only in the nearly identical marginal distributions of intent but also in the high sensitivity and PPV of trauma registrar–coded data.

**Table 4.** Firearm Injury Intent Assigned by Trauma Registrars Compared With Researcher-Adjudicated Intent (2015-2019)

Researcher-adjudicated intent	No. of cases with trauma registrar–coded intent^a^						Intent-specific sensitivity, %
	Unintentional	Assault	Legal intervention	Suicide/self-harm	Undetermined	Total	
Unintentional	142	6	1	5	3	157	90.4
Assault	4	709	4	0	23	740	95.8
Legal intervention	0	2	42	0	0	44	95.5
Suicide/self-harm	1	1	0	123	3	128	96.1
Undetermined	6	26	1	3	10	46	21.7
Total	153	744	48	131	39	1115	NA
Intent-specific PPV of registry data, %	92.8	95.3	87.5	93.9	25.6	NA	NA

Abbreviation: NA, not applicable.

^a^ Brigham and Women’s Hospital trauma registrar data included patient cases with firearm injury incidents meeting our case definition who presented to the emergency department (ED) from 2016 to 2019. The University of Washington–managed Harborview Medical Center trauma registry data included patient cases who presented to the ED from 2016 to 2018. The Massachusetts General Hospital trauma registrar data excluded patient cases with firearm injuries who were treated and released from the ED from 2015 to 2019, following National Trauma Data Bank inclusion guidelines.

## Discussion

In this cross-sectional study of case-level firearm injuries at 3 level I trauma centers, injury intent determined by the research team differed minimally from intent determined by trauma registrars but differed substantially from the intent determined by medical records coders. These findings suggest that the predominant intent-related misclassification problem in discharge data was a tendency to code firearm injuries from assaults as unintentional firearm injuries. This tendency was apparent in *ICD-10-CM* and *ICD-9-CM* coded discharge data, and among patient cases explicitly described in the medical record as having resulted from an altercation, suggesting a more longstanding and insidious problem than can be attributed to the change in *ICD-10-CM* injury intent default guidance alone. Nonetheless, firearm injury intent coding would likely improve, albeit to an indeterminate extent, if *ICD-10-CM* coding instructions defaulted to “assault,” the actuarially most probable intent, and not to “accident” as currently used, especially if coupled with incentives and coding interface modifications that made cases with unambiguous and explicit intent-related language more likely to be coded as noted.

Our findings are consistent with the only other study of which we are aware to compare researcher-adjudicated intent for firearm injuries to *ICD*-coded intent in hospital discharge data. In that study of 122 pediatric patients presenting to a level I trauma center with a firearm injury, 16 of 28 cases *ICD* coded as an unintentional injury in hospital discharge data were classified by the research team as assaults.^14^

Our secondary finding that intent assigned by trauma registrars and by our research team differed minimally suggests that hospitals with trauma registrars already have a reliable source of intent-related information about firearm injuries, especially if registrars code all firearm injury incidents (ie, including those that do not meet the official threshold as trauma cases, as occurred at 2 of our 3 study sites). This close correspondence also suggests that medical records coders, who are more experienced at coding than are research team members, would likely have little trouble characterizing firearm injury intent accurately if incentives were created for them to do so (eg, if supervisors audited the quality of external cause of injury codes).

### Limitations

Several considerations should be borne in mind when interpreting the results of this cross-sectional study. First, there is no gold standard against which to compare researcher-adjudicated intent. Nevertheless, the close correspondence between intent assigned by the research team and by professional coders trained to record detailed information about traumatic injuries (ie, trauma registrars) provides a measure of external validity for our coding rules. Although it is possible that this close agreement resulted from shared coding biases and that other sources of bias are present in the medical records themselves, our finding that many assaults are misclassified as accidents helps explain why a prior study found that patients who were identified in *ICD*-coded discharge data as having suffered an unintentional firearm injury were nearly as likely to be arrested for violent crimes after hospitalization as were patients ICD coded as having suffered assault-related firearm injuries.^15^

Second, study data came from 3 hospitals located in urban settings, all of which were level I trauma centers and are thus not representative of US hospitals. Although we do not have individual-level data from other institutions to directly assess whether the error-producing processes we identified at our study sites underlie *ICD*-coding problems in discharge data more generally, 2 observations suggest that this is plausible. First, the predominant misclassification problem we observed in our discharge data (that many assaults are miscoded as accidents) is the principal distortion others have argued pertains to national data. For example, in 2016, half of firearm injuries in discharge data from the Healthcare Cost and Utilization Project Nationwide Emergency Department Sample were *ICD* coded as unintentional firearm injuries and 43% were coded as assaults, whereas estimates based on more credible sources of intent found that less than 20% were unintentional injuries and more than 70% were assaults.^4^ Second, misclassification problems in our analyses restricted to ED treat-and-release cases were similar in kind and extent to those among admitted cases, suggesting that intent misclassification is largely independent of injury severity (ie, a potentially salient difference between case mix at our sites and elsewhere).

Third, our study used case-level but not coder-level data. Thus, although we can document and quantify discrepancies at the patient-incident level, we can only speculate about any coder-level practices underlying these discrepancies. We are unable to determine, for example, why approximately one-quarter of firearm injury cases that were explicitly described as assault-related in the medical record were *ICD* coded as unintentional injuries in discharge data. One possible explanation is that three-quarters of these cases were coded by medical records coders who almost always *ICD* code such cases as assaults and one-quarter by coders who, for whatever reason and despite explicit evidence of assault in the narrative, coded these cases as unintentional injuries. It is also possible, although less plausible, that the same coder is accurate three-fourths of the time and defaults to unintentional one-fourth of the time. Future studies that elicit coder-level information about the coding process should try to determine how medical records coder practices foster the misclassifications we have described.

## Conclusions

The findings of this cross-sectional study underscore questions raised by prior work about the accuracy of discharge data as a source of firearm injury intent, and by extension, the validity of findings from studies that have used *ICD*-coded discharge data to describe aspects of intent-specific firearm injuries, such as how many have occurred, the demographic distribution of the people injured, and the medical costs they engender.^15,16,17,18,19,20,21,22,23,24,25^

## Notes

Supplement 1. **eAppendix.** Code Book **eTable 1.** Sensitivity and Positive Predictive Value for Intent-Specific Firearm Injuries in Hospital Discharge Data and Trauma Registrar–Coded Data, by Study Site, *ICD-10* vs *ICD-9* Study Period, and Patient Disposition **eTable 2.** Illustrative Narrative Text From the Electronic Medical Record for Different Types of Researcher-Adjudicated Assault Cases and the Corresponding Distribution of *ICD-10-CM* Codes in Discharge Data

Supplement 2.
Data Sharing Statement

## References

[1] US Centers for Disease Control and Prevention National Center for Injury Prevention and Control. Web-Based Injury Statistics Query and Reporting System (WISQARS). 2005. Accessed June 28, 2021. https://www.cdc.gov/injury/wisqars

[2] Barber C. Improving the capacity of hospital ED data systems to track nonfatal firearm injuries. In: Roman J, Cook P, eds. Improving Data Infrastructure to Reduce Firearms Violence. NORC at the University of Chicago; 2021:17-55.

[3] Barber C, Goralnick E, Miller M. The problem with ICD-coded firearm injuries. JAMA Intern Med. 2021;181(8):1132-1133. doi:10.1001/jamainternmed.2021.0382 33779677

[4] Barber C, Cook P, Parker ST. The emerging infrastructure of US firearms injury data. Prev Med. Published online July 5, 2022. doi:10.1016/j.ypmed.2022.107129 35803350

[5] NORC at University of Chicago. Gun Violence Data Infrastructure Report. 2021. Accessed October 25, 2022. https://www.norc.org/Research/Projects/Pages/expert-panel-on-firearms-data-infrastructure.aspx

[6] Weiss R, He C, Gise R, Parsikia A, Mbekeani JN. Patterns of pediatric firearm-related ocular trauma in the United States. JAMA Ophthalmol. 2019;137(12):1363-1370. doi:10.1001/jamaophthalmol.2019.3562 31600369 PMC6802038

[7] Chikani V, Brophy M, Vossbrink A, . Racial/ethnic disparities in rates of traumatic injury in Arizona, 2011-2012. Public Health Rep. 2016;131(5):704-710. doi:10.1177/0033354916663491 28123211 PMC5230812

[8] Izadi SN, Patel N, Fofana D, . Racial inequality in the trauma of women: a disproportionate decade. J Trauma Acute Care Surg. 2020;89(1):254-262. doi:10.1097/TA.0000000000002697 32251262

[9] Tobon M, Ledgerwood AM, Lucas CE. The urban injury severity score (UISS) better predicts mortality following penetrating gunshot wounds (GSW). Am J Surg. 2019;217(3):573-576. doi:10.1016/j.amjsurg.2018.09.013 30292327

[10] Asmar S, Bible L, Vartanyan P, . Firearm-related injuries: a single center experience. J Surg Res. 2021;265:289-296. doi:10.1016/j.jss.2021.03.058 33964639

[11] Feldman KA, Tashiro J, Allen CJ, . Predictors of mortality in pediatric urban firearm injuries. Pediatr Surg Int. 2017;33(1):53-58. doi:10.1007/s00383-016-3984-0 27682469

[12] Choi PM, Hong C, Bansal S, Lumba-Brown A, Fitzpatrick CM, Keller MS. Firearm injuries in the pediatric population: a tale of one city. J Trauma Acute Care Surg. 2016;80(1):64-69. doi:10.1097/TA.0000000000000893 26491805

[13] US Centers for Disease Control and Prevention National Center for Injury Prevention and Control. National Violent Death Reporting System (NVDRS) Coding Manual. Revised April 22, 2021. Accessed October 25, 2022. http://www.ncdsv.org/images/CDC_NatlViolentDeathReportingSystemCodingManual_2003.pdf

[14] Donnelly KA, Badolato GM, Goyal MK. Determining intentionality of pediatric firearm injuries by International Classification of Disease code. Pediatr Emerg Care. 2022;38(1):e306-e309. doi:10.1097/PEC.0000000000002272 33105466

[15] Rowhani-Rahbar A, Fan MD, Simonetti JA, . Violence perpetration among patients hospitalized for unintentional and assault-related firearm injury: a case-control study and a cohort study. Ann Intern Med. 2016;165(12):841-847. doi:10.7326/M16-1596 27750282

[16] Patel SJ, Badolato GM, Parikh K, Iqbal SF, Goyal MK. Sociodemographic factors and outcomes by intent of firearm injury. Pediatrics. 2021;147(4):e2020011957. doi:10.1542/peds.2020-011957 33782104

[17] Peek-Asa C, Butcher B, Cavanaugh JE. Cost of hospitalization for firearm injuries by firearm type, intent, and payer in the United States. Inj Epidemiol. 2017;4(1):20. doi:10.1186/s40621-017-0120-0 28721637 PMC5515719

[18] Schnippel K, Burd-Sharps S, Miller TR, Lawrence BA, Swedler DI. Nonfatal firearm injuries by intent in the United States: 2016-2018 hospital discharge records from the healthcare cost and utilization project. West J Emerg Med. 2021;22(3):462-470. doi:10.5811/westjem.2021.3.51925 34125015 PMC8203029

[19] Kaufman EJ, Wiebe DJ, Xiong RA, Morrison CN, Seamon MJ, Delgado MK. Epidemiologic trends in fatal and nonfatal firearm injuries in the US, 2009-2017. JAMA Intern Med. 2021;181(2):237-244. doi:10.1001/jamainternmed.2020.6696 33284327 PMC7851729

[20] Kalesan B, Zuo Y, Xuan Z, . A multi-decade joinpoint analysis of firearm injury severity. Trauma Surg Acute Care Open. 2018;3(1):e000139. doi:10.1136/tsaco-2017-000139 29766128 PMC5887778

[21] Avraham JB, Frangos SG, DiMaggio CJ. The epidemiology of firearm injuries managed in US emergency departments. Inj Epidemiol. 2018;5(1):38. doi:10.1186/s40621-018-0168-5 30318556 PMC6186529

[22] Coupet E Jr, Huang Y, Delgado MK. US emergency department encounters for firearm injuries according to presentation at trauma vs nontrauma centers. JAMA Surg. 2019;154(4):360-362. doi:10.1001/jamasurg.2018.4640 30673067 PMC6484801

[23] Kalesan B, Siracuse JJ, Cook A, Prosperi M, Fagan J, Galea S. Prevalence and hospital charges from firearm injuries treated in US emergency departments from 2006 to 2016. Surgery. 2021;169(5):1188-1198. doi:10.1016/j.surg.2020.11.009 33384161

[24] Cook A, Osler T, Hosmer D, . Gunshot wounds resulting in hospitalization in the United States: 2004-2013. Injury. 2017;48(3):621-627. doi:10.1016/j.injury.2017.01.044 28173921

[25] Gani F, Sakran JV, Canner JK. Emergency department visits for firearm-related injuries in the United States, 2006–14. Health Aff (Millwood). 2017;36(10):1729-1738. doi:10.1377/hlthaff.2017.0625 28971917

