# Supplementary material for: Assessment of the Accuracy of Firearm Injury Intent Coding at 3 US Hospitals
Source: JAMA Netw Open. 2022 Dec 13;5(12):e2246429. doi: 10.1001/jamanetworkopen.2022.46429 (PMC9856424; doi:10.1001/jamanetworkopen.2022.46429)
Supplement: Supplement 1. — eAppendix. Code Book eTable 1. Sensitivity and Positive Predictive Value for Intent-Specific Firearm Injuries in Hospital Discharge Data and Trauma Registrar–Coded Data, by Study Site, ICD-10 vs ICD-9 Study Period, and Patient Disposition eTable 2. Illustrative Narrative Text From the Electronic Medical Record for Different Types of Researcher-Adjudicated Assault Cases and the Corresponding Distribution of ICD-10-CM Codes in Discharge Data [file jamanetwopen-e2246429-s001.pdf]

Supplementary Online Content

Miller M, Azrael D, Yenduri R, et al. Assessment of the accuracy of firearm injury intent coding at 3 US hospitals. *JAMA Netw Open*. 2022;5(12):e2246429. doi:10.1001/jamanetworkopen.2022.46429

eAppendix. Code Book

eTable 1. Sensitivity and Positive Predictive Value for Intent-Specific Firearm Injuries in Hospital Discharge Data and Trauma Registrar–Coded Data, by Study Site, *ICD-10* vs *ICD-9* Study Period, and Patient Disposition

eTable 2. Illustrative Narrative Text From the Electronic Medical Record for Different Types of Researcher-Adjudicated Assault Cases and the Corresponding Distribution of *ICD-10-CM* Codes in Discharge Data

This supplemental material has been provided by the authors to give readers additional information about their work.

© 2022 Miller M et al. *JAMA Network Open*.

eAppendix. Code Book

Our coding manual drew on definitions and rules for coding intent from codebooks for the National Violent Death Reporting System (NVDRS), which were then refined iteratively by team members as we reviewed cases.

Legend: Variable names are noted in **ALLCAPS**. Words that are in all caps but are not bold and have an asterisk in them (e.g., *PHRASE), indicate that a repeating variable or parallel variables will appear directly below.

1. **Case Definition Variables**: Review the relevant portions of the electronic health record and code three variables to establish whether the case meets the project **case definition** as an initial encounter for an injury resulting from a projectile fired from a firearm:

    a. **GSW**

    Definition: Injury resulted from a traditional firearm projectile (non-traditional projectile defined below) fired from a firearm

    Response Options:
    1 Yes
    0 No/unknown

    b. **NOTGSW** (if GSW=0)

    Definition: Type of injury or condition if not a firearm projectile injury

    Response Options:
    1 Not a firearm (e.g., BB gun, air rifle, nail gun, paint gun, Verey pistol, flare gun, pellet gun etc.)
    2 Non-projectile firearm injury (e.g., pistol whipping, injury to thumb webbing from pistol slide, burn from spent casing)
    3 Non-traditional projectile (e.g., beanbag ammo, rubber bullet)
    4 Other (medical condition, other type of injury)
    9 Unknown

    c. **ARRIVAL**

    Definition: How did the patient arrive at the ED?

    Response Options:
    1 Ambulance or Airlift (EMS)
    2 Law Enforcement
    3 Transfer from Another Hospital
    4 Other

    d. **FIRST**

    Definition: Was this the first episode of care? The objective of the project is to determine the number of hospital-treated gunshot injuries that occur by intent. To that end, to avoid the double-counting that would result if multiple visits for the same injury were treated as cases, we code only those types of presentation listed below as first encounters.

    Response Options:

© 2022 Miller M et al. *JAMA Network Open*.

1 Yes (first episode of care following injury; includes ED presentation, transfer from another ED, admission from ED. If someone has never sought care and is now presenting for wound infection, that counts as first episode)
0 No (e.g., subsequent care after first ED visit/transfer/admission, e.g., for suture removal, wound check, wound infection, sequelae of injury, late effects. If a person visited a hospital earlier, walked out and visited another hospital for the same injury, the second visit=0 for first episode of care)
9 Unknown

Continue with remaining steps only if GSW=1 and FIRST=1

2. **Intent Classification**: (If GSW=1 and FIRST=1) Enter five variables to classify intent and indicate the specific phrases that led to that classification.

a. INTENT

Definition: Classification of firearm injury intent.

See detailed definition of each intent in Coding Guidance, p. 7.

1	SUICIDE/INTENTIONAL SELF-HARM
2	ASSAULT/INTERPERSONAL VIOLENCE
3	ACCIDENT/UNINTENTIONAL
4	LEGAL INTERVENTION
8	UNABLE TO DETERMINE DUE TO CREDIBLE CONFLICTING EVIDENCE
9	UNKNOWN – NOT ENOUGH INFORMATION

b. ***PHRASE** Five free-text fields into which abstractor types verbatim the key, specific phrases from the Electronic Health Record (EHR) that provide information about intent. Include all statements relevant to intent even if they do not support the intent type ultimately coded. For each of the free text fields, indicate the location of the intent information, as described below. Some examples of phrases:

- "Shot on street while walking with friend; friend also shot and died."
- "Shot by unknown assailant."

IntentLocation1
Definition: First Location of intent information in HER
Response Options:
1 Outside Report: records from sources such as ambulance, airlifts
2 ED Notes
3 Inpatient Notes
4 Discharge Summary
5 Other

TextField1
Definition: Please copy the relevant phrases from the above location into the box.

© 2022 Miller M et al. *JAMA Network Open*.

IntentLocation2
Definition: Second Location of intent information in EHR.
Response Options:
1 Outside Report: records from sources such as ambulance, airlifts
2 ED Notes
3 Inpatient Notes
4 Discharge Summary
5 Other

TextField2
Definition: Please copy the relevant phrases from the above location into the box.

IntentLocation3
Definition: Third Location of intent information in EHR.
Response Options:
1 Outside Report: records from sources such as ambulance, airlifts
2 ED Notes
3 Inpatient Notes
4 Discharge Summary
5 Other

TextField3
Definition: Please copy the relevant phrases from the above location into the box.

IntentLocation4
Definition: Fourth Location of intent information in EHR.
Response Options:
1 Outside Report: records from sources such as ambulance, airlifts
2 ED Notes
3 Inpatient Notes
4 Discharge Summary
5 Other

TextField4
Definition: Please copy the relevant phrases from the above location into the box.

IntentLocation5
Definition: Fifth Location of intent information in EHR.
Response Options:
1 Outside Report: records from sources such as ambulance, airlifts

© 2022 Miller M et al. *JAMA Network Open*.

2 ED Notes
3 Inpatient Notes
4 Discharge Summary
5 Other

TextField5
Definition: Please copy the relevant phrases from the above location into the box.

c. AXIS* (only if INTENT=8) *Definition:* Which intent types conflict? For example, if the injury was clearly self-inflicted but unclear whether intentional or unintentional, code AXIS1=”1, suicide/intentional self-harm” and AXIS2=”3, accident.”
AXIS1 (use INTENT code list)
AXIS2 (use INTENT code list, or choose “7” if there is more than one alternate intent being considered beyond AXIS1)

4. **Circumstance leading to shooting**: Code the circumstance of the incident in one of four intent-specific *CIRC Intent-specific circumstance associated with the shooting. *Code just one variable below, or code none if INTENT= 8 or 9 (unknown).*

SUICCIRC (if INTENT=1)
Definition: Type of intentional self-harm.
Response Options:
1 Suicide attempt
2 Non-suicidal self-harm
9 Unknown

VIOLCIRC (if INTENT=2)
Definition: Precipitating circumstance of the Assault/Interpersonal Violence.
Response Options:
1 Intimate partner or family violence
2 Felony-type crime (occurred during the commission of a, or in reference to, a non-IPV/DV felony, like robbery, drug trade, car-jacking)
3 Gang-related (other than IPV/DV or felony-type)
4 Argument (e.g., drunk people arguing at party, road rage, revenge over sleight)
5 Law enforcement officer injured on active duty
7 Other precipitating circumstance (e.g., psychotic shooting, justifiable)
9 Unknown

ACCIDCIRC (if INTENT=3)
Definition: Context of Accident/Unintentional shooting.
Response Options:
1 Playing/fooling around with gun
2 Hunting or target shooting accident
3 Cleaning gun
4 Holstering/unholstering gun (includes removing/returning to holster,pocket, waistband)
5 Other functional handling (e.g. while transporting, showing gun)

© 2022 Miller M et al. *JAMA Network Open*.

7 Other unintentional discharge
9 Unknown

LEGALCIRC (if INTENT=4)

Definition: Type of circumstance that brought police and pt. together.

Response Options:

1 Mental health call (e.g., suicide threat, naked guy yelling in street)
2 Intimate partner/family violence or disturbance (other than mental health call)
3 Felony-type crime
4 Traffic stop
7 Other
9 Unknown

5. **Relationship of Shooter to Patient**

a. **SELF**

Definition: Shot by self, someone else, or unknown

Response Options:

0 Someone else
1 Self
9 Unknown

b. **RELATION**

Definition: Relationship of shooter to patient, if shot by another person (e.g., patient was shot by x)

Response Options:

1 Current or former intimate partner
2 Other family
3 Acquaintance (neighbor, classmate, friend of a friend)
4 Police
5 Stranger
6 Unspecified (unknown)
7 Known shooter

6. <u>ADDITIONAL CONTEXT CODES</u>:

a. **MULTIVICT**

Definition: more than one person was shot during the incident

Response Options:

0 No
1 Yes

b. **MULTISHOTS**

© 2022 Miller M et al. *JAMA Network Open*.

Definition: Multiple shots (either multiple bullet wounds or heard multiple shots). If multiple GSWs from one bullet (e.g. single bullet causing an entry and exit wound), code as no; if multiple gunshot wounds and it is unknown whether from a single or multiple shots, code as yes.

Response Options:
0 No or unknown
1 Yes

c. **WITHHOLD**

Definition: Patient appears to be withholding information (e.g., pt refuses to speak; pt uses some version of "I was walking along, heard some pops, and next thing I knew…")

Response Options:
0 No or unknown
1 Yes

d. **2STORIES**

Definition: Conflicting stories or evidence point to conflicting intent types (regardless of the credibility of that evidence).

Response Options:
0 No or unknown
1 Yes

e. **DRIVEBY**

Definition: Shooter was riding in/on a vehicle (car, bicycle, truck) when he/she shot patient and patient was not in the car with the shooter.

Response Options:
0 No or unknown or not applicable (for example, if the patient shot themselves)
1 Yes

f. **PLACE**

Definition: Where pt. was when the shooting occurred.

Response Options:
1 House, apartment, including driveway, porch, yard, garage
2 Street, sidewalk
3 Motor vehicle (victim was sitting in, driving, getting in or out of MV, regardless of where vehicle is located)
4 Parking lot/public parking garage
5 Public transportation or station (e.g., bus, train, plane, airport, depot, taxi)
6 Bar, nightclub
7 Service station
8 Liquor store
9 Hotel/motel
10 Other commercial establishment
11 Public institution (e.g., hospital, prison, house of worship, social service agency, etc.)
12 Sports or athletic area (e.g., stadium, baseball field, gymnasium, recreation center)
13 Park, playground, public use area

© 2022 Miller M et al. *JAMA Network Open*.

14 Farm
15 Natural area (e.g., field, river, beach, woods)
16 Shooting area (e.g., shooting range, sportsman club)
17 Party, unspecified location
77 Other
99 Unknown

g. **ACTIVITY**

Definition: What activity was the patient doing when they were shot? (These are *ICD* activity codes)

Response Options:
1 While engaged in sports activity
2 While engaged in leisure activity
3 While working for Income
4 While engaged in other types of work (e.g., chores, school)
5 While resting, sleeping, eating, or engaging in other vital activities
6 While involved in altercation or fight
7 While engaged in other specified activities
8 While engaged in unspecified activities

h. **Location of Injury**

Definition: Where are the GSW(s) on the body? (each of these are separate 0/1 variables)
HEAD
FACE
NECK
UPPEREXT – hand, arm, shoulder (LUE, RUE)
BACK- spine
CHEST - thorax
ABDOMEN- flank
LOWEREXT – leg, foot, buttocks

i. **DISPOSITION**

Definition: Describe the final outcome of the hospital stay We code for, transfers, admissions and deaths so that they can be included or omitted as necessary in counts (e.g., if one were counting the number of gunshot injuries that were treated at hospitals, it would be appropriate to include injuries that resulted in death; this would not be the case if the objective were to count non-fatal injuries only).

Response Options:
1 Discharged from ED (to home, police custody, etc.)
2 Admitted to hospital associated with ED and discharged from hospital
3 Died
4 Transferred to another acute care hospital
7 Other
9 Unspecified

© 2022 Miller M et al. *JAMA Network Open*.

j. **LOCDEATH** (If Outcome = 3, Died)

Definition: Where patient died.

Response Options:

1 Dead on arrival
2 Emergency department
3 Inpatient unit
4 Other/unspecified

CODING GUIDANCE (INTENT)

Intentional Self-harm (INTENT=1)

Any self-inflicted injury resulting from the purposeful use of force against oneself. This category includes both suicide attempts and non-suicidal intentional self-harm. The variable SELFCIRC allows the coder to distinguish between suicide attempts and non-suicidal self-harm, when known.)

Use of the phrase "self-inflicted" alone can generally be interpreted to mean "intentionally self-inflicted" if the record does not indicate it was an accident.

The fact that an intentional, self-inflicted act was undertaken while under the influence of a mental illness or mind-altering drug does not change the classification.

Russian roulette is classified as Suicide/Intentional Self-harm.

"[Attempted] suicide by cop" should be classified as Legal Intervention, not Suicide/Intentional Self-harm, if the shot was taken by police.

If a person shoots his or herself while fleeing from, or engaged in a conflict with, police, classify the act as Suicide/Intentional Self-harm, not Legal Intervention.

Examples of SUICIDE/INTENTIONAL SELF-HARM (followed by how SUICCIRC should be coded for that case):

- "Pt attempted suicide with shotgun blast to head." *SUICCIRC=1 (Suicide Attempt)*
- "GSW to head after long struggle with bipolar disorder." *SUICCIRC=1*
- "Pt shot self in front of his ex-girlfriend after attempted reconciliation." *SUICCIRC=1*
- "Pt shot himself in the heart; was due to be sentenced the next day and said he would not return to prison." *SUICCIRC=1*
- "Police were engaged in motor vehicle chase after pt robbed a convenience store. Pt pulled over and shot self in the head." *SUICCIRC=1*
- "Pt had been drinking at a party and was playing Russian roulette." SUICCIRC=1
- "Gun found on scene, suicide note found." SUICCIRC=1
- "Pt under the influence of meth shot knee because he was angry about pain in his knee." *SUICCIRC=2 (Non-suicidal self-harm)*
- "Pt with history of schizophrenia shot herself in abdomen in attempt to abort pregnancy." SUICCIRC=2
- "Self-inflicted gunshot wound" (provided there is no evidence that it was accidental) SUICCIRC=3 (Unknown if suicidal or non-suicidal)
- "impulsive" act of self-inflicted gsw in setting of frustration from dispute. SUICCIRC=3

Assault/interpersonal violence (INTENT=2)

© 2022 Miller M et al. *JAMA Network Open*.

For this project, we will default to assigning all cases to assault/interpersonal violence if the case does not meet the criteria for intentional self-injury, unintentional injury, legal intervention, or injury of unknown intent, per the guidance provided here.

Cases with more than one victim (VICTIMS=1) or multiple gun shots sustained or heard (MULTIPLE=1) should be coded as assault, even if no additional information is available.

Code shootings with information about any of the following as assault: shot by (person x), shot while (activity x), or shot at (location x).

Code as assault cases that don't have any circumstantial information, but mention the terms: assault, assailant, perpetrator, victim, community violence, or referrals to a violence recovery program.

If no circumstantial information about the shooting is present, but the police are treating the incident as a homicide, looking for suspect, or if someone was arrested for the incident, code as assault.

NB: for cases in which there is sufficient circumstantial information about the shooting incident provided to code assault:

1) the gunshot wound itself need not have been intentionally inflicted to be classified as assault, but the use of the firearm to threaten, harm, or defend against another person must be. For example, say someone shoots up a building to threaten its residents, but shots penetrate the wall and a resident is unintentionally injured by a bullet. The case will be classified as assault even though the injury was unintended.
2) Gunshot injuries inflicted by a law enforcement officer are separately classified (as below).

Examples of cases to be coded as assault/interpersonal violence include:

- "Pt shot by clerk when he was attempting to rob a liquor store"
- "Bystander was unintentionally shot during gunfight between two others"
- "Pt was shot during a drug deal"
- "Pt was a police officer who was shot while attempting to serve a search warrant"
- "Police are treating the case as an attempted murder"
- "Pt. states was walking home when someone started shooting at him; cannot identify person"
- "Involved in altercation, sustained GSW"
- "Victim of community violence" "Victim of gun violence"
- Mention of assault, assailant (e.g., "shot by unknown assailant")
- "The police suspect the shooting was gang-related"
- "Pt reports not having a history with perpetrator"
- "Leaving a party when unknown person began shooting; doesn't believe she was the intended target"
- "Pt states he was walking along and heard a shot and realized he was hit"
- "Pt was getting out of his car when he was shot"
- "Pt was shot at while she was standing with friends at the park"
- "Shot from a passing car"
- "GSW to left shoulder; pt states he wasn't the intended target"
- No information about incident, but referrals or discharge planning implies violence (e.g., "Pt referred to violence interruption program," "Pt believes it will be safe to return to his neighborhood"

© 2022 Miller M et al. *JAMA Network Open*.

- “Pt reports he was drinking alcohol and he only heard one shot.”
- “Pt was standing and was shot in the RLQ with a 22 caliber gun.”
- “Pt presents with multiple gunshot wounds to the head, right lower extremity, and chest.”

Accident/unintentional (INTENT= 4)

Injury resulted from the unintentional discharge of a firearm (e.g., shooter mistakenly pulled trigger) or from the intentional (but not violence-related) discharge of a gun if the shooter was unaware that an injury would occur (e.g., pt shot when a fellow hunter was swinging on prey; teenager shot when fooling around with a gun he thought was unloaded). Accidents can be self-inflicted or inflicted by another person. If a patient unintentionally shot self when using a gun to defend against or threaten another person, code that as an accident. If someone else was injured, code it as assault/interpersonal violence).

Examples:

- Celebratory firing that was not intended to frighten, control, or harm anyone (e.g., “Neighbors were shooting in celebration on 4th of July; pt was standing on a balcony and was hit.”)
- Child under age 6 shoots self or another person (too young to be considered intentional)
- “Pt. stumbled and shot foot while in yard with gun drawn to investigate suspicious noises”
- “Two youths were playing ‘quick draw’ with pistols they thought were unloaded.”

Indistinct language – Sometimes, given the context, the intent is implied but not explicitly stated. When a shooting was stated as occurring while the shooter was cleaning or repairing a gun, target shooting, engaged in hunting or pest control, playing with a gun (other than in a Russian roulette type game or a threatening game), or during routine handling, intent can be noted as “accident” if no evidence exists to the contrary.

Examples:

- “Pt shot during a hunting incident”
- “Pt sustained GSW while pulling rifle from truck bed of her pickup”
- “GSW to left shoulder while in woods behind outdoor shooting range”
- “GSW while dismounting a tree blind“
- “Pt was injured while cleaning his handgun”
- “Pt. and friends were horsing around with a gun”
- “patient’s friend was showing his gun to the patient and it discharged”
- “gun misfired while patient’s sister was trying to remove the gun from patient to prevent him from harming himself”

Legal Intervention (INTENT=4)

Code shootings as legal intervention if the shooter was a sworn officer who was acting in the line of duty. A “*sworn officer*” includes someone employed by a public agency with statutory authority to use lethal force, including a police officer, sheriff, sheriff’s deputy, corrections officer, federal law enforcement officer (e.g. FBI, secret service, drug enforcement agent, ATF agent), military police, animal control officer. It does not include a private security guard. An incident occurred “*in the line of duty”* if the officer was on duty (provided the case was not a clear murder, such as an on-duty officer who murders his or her spouse) or if an off-duty officer interceded as an officer. Some shootings in the line of duty may seem unjustifiable; coding them as legal interventions does not imply that they were necessarily justifiable, simply that they were related to legal intervention. Finally, in some cases an off-duty officer is personally the victim of a crime and responds by shooting the alleged offender. These cases

will be coded as assault/violence-related, not legal intervention (e.g., off duty officer encounters a person who is trying to break into officer's home and shoots him).
In unusual circumstances, some "legal intervention" cases will be accidental, not interpersonal violence-related, but these should still be coded as "legal intervention" (e.g., pt was shot when a police officer shot at a bear).

Examples:

- Pt. shot by police during an attempted carjacking.
- Pt had someone at gunpoint in a bar. Off-duty officer who was working security announced himself as an officer and shot pt when pt refused to drop the gun.
- Pt was a police officer who was hit by "friendly fire" from another officer during a foot chase with a suspect.
- Pt was shot while attempting to escape from prison.
- Pt was shot during a routine traffic stop when police thought he was reaching for a gun.

Unable to determine due to conflicting evidence (INTENT=8)

Resolvable conflicting cases:
When there is suggestive evidence for more than one intent type and abstractor **can find some basis** for deciding between them.
Example:

- "Pt. on arrival states this was an assault and later stated he'd actually shot himself accidentally and had been too embarrassed to admit it"

Unresolvable conflicting cases:
When there is suggestive evidence for more than one intent type and abstractor **can find no basis** for deciding between them.

Example:

- "Notes from social worker indicate she thinks this may be an accident; notes from EMS state woman shot in DV incident, police arrested husband."

Unknown (not enough information) (INTENT=9)
Code shootings as unknown when nothing is known about the circumstances of the shooting and no information in the record suggests that the injury was the result of an assault. The fact that police were involved does not necessarily mean the case was an assault.
Example:

- "Pt with GSW to the head transferred from Mercy Hospital; circumstances unknown."
- "Pt found in ditch, GSW to chest"
- "Found down in street"
- "Pt who is under house arrest transferred by EMS after GSW to foot under unclear circumstances."
- "Pt and friend report to the ED; friend was reportedly without injuries, but patient had gsw to head."
- "Pt is not sure exactly when the gun shot happened, he does not know who shot him. He states at the time of the shot he "blacked-out". He states he was at home and noticed his left thigh hurt and then he was at a hospital."

© 2022 Miller M et al. *JAMA Network Open*.

- “Pt admitted with GSW to back. Circumstances of shooting remain unclear. Police are involved and interviewed pt in ED.”
- “Pt arrives with a GSW to left arm. Pt declined to answer questions. History limited by patient’s unwillingness to participate.”
- “Pt states he was shot in the abdomen”
- “Pt presents with GSW to the chest. Police are involved.

© 2022 Miller M et al. *JAMA Network Open*.

eTable 1. Sensitivity and Positive Predictive Value for Intent-Specific Firearm Injuries in Hospital Discharge Data and Trauma Registrar–Coded Data, by Study Site, *ICD-10* vs *ICD-9* Study Period, and Patient Disposition

		Sensitivity					Positive Predictive Value				
	N	Unintentional	Assault	Legal Intervention	Self-harm	Undetermined	Unintentional	Assault	Legal Intervention	Self-harm	Undetermined
**TR at MGB**	246	88%	98%	100%	100%	8%	100%	94%	80%	96%	25%
**TR at HMC**	869	91%	95%	94%	95%	26%	92%	96%	89%	93%	26%
**ICD-10 MGB**	358	78%	62%	57%	84%	11%	21%	94%	100%	89%	10%
**ICD-10 HMC**	869	91%	69%	72%	90%	5%	40%	96%	93%	94%	3%
**ICD-10 Fatal (all sites)**	155	86%	61%	63%	87%	17%	17%	91%	100%	100%	13%
**ICD-10 Non-fatal (all sites)**	1072	88%	67%	71%	92%	2%	36%	96%	93%	88%	3%
**ICD-10 ED treat and released (all sites)**	450	94%	68%	67%	89%	5%	34%	95%	100%	100%	4%
**ICD-10 ED to Hospital Admission (all sites**	740	86%	65%	73%	92%	4%	36%	96%	92%	87%	3%
**ICD-9 (only colected at MGB)**	783	89%	67%	69%	74%	8%	44%	90%	90%	77%	22%

© 2022 Miller M et al. *JAMA Network Open*.

eTable 2. Illustrative Narrative Text From the Electronic Medical Record for Different Types of Researcher-Adjudicated Assault Cases and the Corresponding Distribution of *ICD-10-CM* Codes in Discharge Data

Circumstance Category	ICD-coded as Unintentional (N= 234)		ICD-coded as Assault (N= 555)		ICD-coded as Undetermined Intent (N= 40)		Total
**Drive-by**	"male who was the victim of a **drive-by shooting** earlier today when he was shot twice, once in the left hand and wrist and a second time just grazing his left occiput"	37 (23.4)	"Per report pt was involved in a **drive-by shooting** and pt sustained single GSW to the chest."	118 (74.7)	"male from <city> who was hit with a stray bullet at a gas station during a **drive-by shooting**."	3 (1.9)	158 (100.0)
**Assailant**	"He heard 2 shots, one when he was face to face with his **assailant**, one while he was running away."	40 (20.0)	"The patient reports that he was exiting a bus...when an unknown **assailant** fired a single shot to his left thigh"	152 (76.0)	"Per report, patient was standing outside a pizza shop when he was shot by an unknown **assailant** in the shoulder."	8 (4.0)	200 (100.0)
**Altercation**	"He was at a party earlier in the night and was caught in an **altercation** trying to leave the party, at which point some shots were fired."	36 (21.8)	"He was attempting to break up an **altercation** between people that he reports he did not know when he was shot."	124 (75.2)	"Pt did not provide PD with extensive details of assault, notes that he was shot in a vehicle during an **altercation** over drugs."	5 (3.0)	165 (100.0)

*The sum of row totals and the sum of column totals exceeds researcher-adjudicated assaults in Table 1 because cases could have more than one indicator of assault intent.

© 2022 Miller M et al. *JAMA Network Open*.
