# Supplementary material for: Assessment of the Accuracy of Firearm Injury Intent Coding at 3 US Hospitals
Source: JAMA Netw Open. 2022 Dec 13;5(12):e2246429. doi: 10.1001/jamanetworkopen.2022.46429 (PMC9856424; doi:10.1001/jamanetworkopen.2022.46429)
Supplement: Supplement 2. — Data Sharing Statement [file jamanetwopen-e2246429-s002.pdf]

Data Sharing Statement

Miller. Assessment of Accuracy of Firearm Injury Intent Coding at 3 US Hospitals. *JAMA Netw Open*. Published December 13, 2022. doi:10.1001/jamanetworkopen.2022.46429

Data
Data available: No

Additional Information
Explanation for why data not available: Data contain personal health information.
